# Phospholipase D2 controls bone homeostasis by modulating M-CSF-dependent osteoclastic cell migration and microtubule stability

**DOI:** 10.1038/s12276-022-00820-1

**Published:** 2022-08-09

**Authors:** Hyun-Ju Kim, Dong-Kyo Lee, Xian Jin, Xiangguo Che, Sung Ho Ryu, Je-Yong Choi

**Affiliations:** 1 Department of Biochemistry and Cell Biology, Cell and Matrix Research Institute, BK21 Plus KNU Biomedical Convergence Program, Korea Mouse Phenotyping Center, School of Medicine, Kyungpook National University, Daegu, 41944 Republic of Korea; 2 Department of Life Sciences, Pohang University of Science and Technology, Pohang, 37673 Republic of Korea

**Keywords:** Bone, Microtubules

## Abstract

Phospholipase D2 (PLD2), a signaling protein, plays a central role in cellular communication and various biological processes. Here, we show that PLD2 contributes to bone homeostasis by regulating bone resorption through osteoclastic cell migration and microtubule-dependent cytoskeletal organization. *Pld2*-deficient mice exhibited a low bone mass attributed to increased osteoclast function without altered osteoblast activity. While *Pld2* deficiency did not affect osteoclast differentiation, its absence promoted the migration of osteoclast lineage cells through a mechanism involving M-CSF-induced activation of the PI3K–Akt–GSK3β signaling pathway. The absence of *Pld2* also boosted osteoclast spreading and actin ring formation, resulting in elevated bone resorption. Furthermore, *Pld2* deletion increased microtubule acetylation and stability, which were later restored by treatment with a specific inhibitor of Akt, an essential molecule for microtubule stabilization and osteoclast bone resorption activity. Interestingly, PLD2 interacted with the M-CSF receptor (c-Fms) and PI3K, and the association between PLD2 and c-Fms was reduced in response to M-CSF. Altogether, our findings indicate that PLD2 regulates bone homeostasis by modulating osteoclastic cell migration and microtubule stability via the M-CSF-dependent PI3K–Akt–GSK3β axis.

## Bone disease: Possible treatment to rebalance bone remodeling

A signaling protein that regulates bone resorption may prove a useful target in treating skeletal conditions such as osteoporosis and rheumatoid arthritis. Bone is synthesized by cells called osteoblasts, while osteoclasts trigger bone resorption, keeping the skeleton healthy. Imbalances in this recycling process are common in bone disorders. Je-Young Choi and Hyun-Ju Kim at Kyungpook National University in Daegu, South Korea, and co-workers demonstrated that phospholipase D2 (PLD2), a membrane protein, directly regulates bone resorption in mice. Mice without the *Pld2* gene had increased osteoclast activity, resulting in low bone mass. The absence of PLD2 promotes the migration of osteoclasts via a particular signaling pathway. This increased the organization of microtubules, polymers that help form the cytoskeleton. The results suggest that regulating PLD2 activity could form the basis of a future treatment method.

## Introduction

Skeletal homeostasis is strongly controlled by the dynamic coordination of bone-degrading osteoclasts and bone-synthesizing osteoblasts^1–4^. Osteoclasts are multinuclear cells responsible for resorbing the calcified bone matrix. The development of these polykaryons is a multistage biological process that comprises the proliferation, differentiation, migration, and maturation of macrophage-monocyte lineage precursors. The osteoclast differentiation process requires the activation of osteoclastogenic signaling cascades through the binding of receptor activator of nuclear factor-κB ligand (RANKL) to its receptor, RANK^5–8^. On the other hand, precursor proliferation, migration, and cytoskeletal organization require the activation of signals through macrophage colony-stimulating factor (M-CSF) and its receptor, c-Fms^9–12^.

The degradation of bone matrix by osteoclasts depends on the organization of their actin cytoskeleton, resulting in polarization. When osteoclasts contact bone, they become polarized, forming a unique adhesive structure, F-actin-rich ring or sealing zone, which surrounds the resorption area, and this event requires intact microtubule integrity^13,14^. Microtubules are the major components of the eukaryotic cytoskeleton and comprise α- and β-tubulin protein subunits. These polymers play an important role in various cellular events, including polarization, migration, vesicular trafficking, and the maintenance of cell shape. The importance of the tight modulation of microtubules in bone resorption has been well documented in numerous studies^15–20^. Notably, acetylated tubulin, a marker of stable microtubules, plays a critical role in generating actin rings or sealing zones in mature osteoclasts. The stabilization of the actin ring belt correlates with increased levels of tubulin acetylation, and this process is regulated by the Rho–mDia2–HDAC6 pathway^15^. The Akt–GSK3β axis also governs osteoclast microtubule stability and bone resorption. The specific deletion of Akt1 and Akt2 in mature osteoclasts resulted in increased bone mass due to impaired actin ring formation and bone resorption, revealing the essential role of Akt signaling in osteoclast bone-resorbing activity^19^.

Members of the phospholipase D (PLD) family are enzymes that cleave phosphatidylcholine, the major membrane phospholipid, into the bioactive lipid phosphatidic acid and choline^21–23^. Two main PLD isoforms (PLD1 and PLD2) share ~50% sequence homology and have similar structures but differ in their subcellular localization. PLD1 is present in vesicular organelles, such as endosomes, autophagosomes, and lysosomes, whereas PLD2 is mainly localized in the plasma membrane. PLD1 and PLD2 participate in several cellular functions, such as cell proliferation, migration, vesicle trafficking, and cytoskeletal organization. Interestingly, a previous study using *Pld1* or *Pld2* knockout mice demonstrated that PLD1 and PLD2 play distinct roles in mast cell activation by regulating microtubule formation^24^. This study further showed that PLD2 acts as a negative regulator of the organization of the mast cell cytoskeleton. In bone metabolism, several studies have revealed that PLD isoforms are involved in modulating bone cells^25–28^. Our recent study reported that *Pld1*-deficient mice displayed reduced bone mass attributed to impaired osteoblastogenesis and increased osteoclastogenesis^26^. Another study showed that PLD1, but not PLD2, enhances the osteoblast-mediated mineralization process^25^. Although PLD1 plays a role in bone metabolism, the function of PLD2 in skeletal tissue homeostasis remains uncertain.

In this study, employing *Pld2*-deficient mice, we demonstrate that PLD2 plays a vital role in bone homeostasis as a negative regulator of osteoclastic bone resorption. Ablation of *Pld2* accelerated cell migration and microtubule acetylation and increased the actin ring size, leading to an osteopenic phenotype in mice. We further show that PLD2 functions in osteoclasts through the M-CSF-mediated PI3K–Akt–GSK3β signaling pathway.

## Materials and methods

### Reagents

Recombinant human M-CSF and RANKL were acquired from R&D Systems (Minneapolis, MN, USA). LY294002 and PD98059 were purchased from Cayman Chemical (Ann Arbor, MI, USA), and MK2206 was purchased from Selleck Chemicals (Selleck Chemicals, TX, USA). Antibodies against Akt, ERK, JNK, p38, IκBα, PI3K, and PLD2 and phospho-specific antibodies for Akt, ERK, JNK, and p38 were obtained from Cell Signaling Technology (Beverly, MA, USA). Antibodies against c-Fms, PLD2, GSK3β, and phospho-GSK3β were purchased from Santa Cruz Biotechnology (Santa Cruz, CA, USA). Anti-tubulin and anti-acetylated tubulin antibodies were purchased from Sigma-Aldrich (St. Louis, MO, USA).

### Mice

*Pld2* knockout mice were produced as reported previously^29^ and kept on a C57BL/6 background. The animals were housed in the animal facility at Kyungpook National University. All studies were approved by the Committee on the Ethics of Animal Experiments of Kyungpook National University (approval number: KNU-2019-0038).

### Microcomputed tomography (micro-CT)

Mouse femurs were collected and fixed with 4% paraformaldehyde for 24 h. The microarchitectural properties of the femur were obtained using a micro-CT system (eXplore Locus SP, GE Healthcare, Waukesha, WI, USA). A region between 0.7 and 2.3 mm below the growth plate was set as the region of interest. Next, the femurs were scanned with a micro-CT calibrated to X-ray energy settings of 80 kV and 80 μA and an effective detector pixel size of 0.008 mm. Finally, the bone parameters were determined using eXplore MicroView v.2.2 software provided with the micro-CT system.

### Histology and histomorphometry

For dynamic bone histomorphometric analysis, 7-week-old mice were injected intraperitoneally with calcein green (10 mg/kg) and alizarin red (20 mg/kg) 6 and 2 days before sacrifice, respectively, as described in a previous study^30^. The tibiae were isolated, fixed in 4% paraformaldehyde, and embedded in methyl methacrylate. The embedded tibiae tissues were cut into 6-μm-thick sections using a Leica RM2165 rotary microtome with a tungsten blade (Leica Microsystems, Germany) and then imaged under a fluorescence microscope (Leica Microsystems, Germany). For von Kossa staining analysis, the lumbar vertebrae were fixed and embedded in methyl methacrylate. After that, 6-µm-thick vertebrae sections were stained with von Kossa reagent. To evaluate osteoclast parameters, the tibiae were harvested and fixed in 4% paraformaldehyde, followed by decalcification in 10% EDTA for 4 weeks at 4 °C. The specimens were dehydrated and embedded in paraffin. The paraffin sections were then stained for tartrate-resistant acid phosphatase (TRAP) to identify osteoclasts. Bone histomorphometric analyses were conducted using the Bioquant OSTEO II program (Bio-Quant, Inc., Nashville, TN, USA).

### Osteoclast culture

Murine bone marrow-derived macrophages (BMMs) and osteoclasts were prepared as described previously. In brief, bone marrow was extracted from the long bones of 8–9-week-old mice, and red blood cells were lysed for 2 min at room temperature. The cells were then plated in a Petri dish and cultured for 3–4 days in α-minimal essential medium (α-MEM) containing 10% fetal bovine serum and 10% CMG 14-12 cell culture medium as the M-CSF source^31^. The attached cells were lifted and used as osteoclast precursor cells (BMMs). BMMs were then seeded at a density of 5 × 10^3^ per well in a 96-well cell culture plate and incubated in α-MEM supplemented with M-CSF and RANKL for osteoclast generation. Osteoclasts were fixed using 4% paraformaldehyde after 4–5 days in culture and stained with TRAP solution containing 0.1 mg/ml naphthol AS-MX phosphate and 0.3 mg/ml Fast Red Violet.

### Proliferation and apoptosis assays

Proliferation assays were conducted using the Cell Proliferation Biotrak ELISA kit (Amersham, GE Healthcare Life Sciences). BMMs were cultured in a 96-well plate at 5 × 10^3^ cells per well in α-MEM supplemented with M-CSF at various concentration for 3 days. After the cells had been incubated with 0.1% bromodeoxyuridine (BrdU) at 37 °C for 4 h, the amount of BrdU incorporation into the cellular DNA was determined by measuring the absorbance at 450 nm. For the apoptosis assay, BMMs were plated at a concentration of 5 × 10^3^ cells per well in a 96-well plate and cultured in α-MEM containing M-CSF (30 ng/ml) for 3 days. This assay was performed using the Cell Death Detection ELISA Plus kit (Roche, Mannheim, Germany) according to the manufacturer’s instructions.

### Migration assay

Migration assays were performed using a Transwell migration assay kit (Corning, Corning, NY, USA). First, BMMs (2 × 10^5^ cells per well) or preosteoclasts (2 × 10^5^ cells per well) were seeded in the upper chamber in 100 μl of serum-free α-MEM, and M-CSF (50 ng/ml) was added to the lower chamber in 600 μl of serum-free α-MEM. After 16 h of incubation at 37 °C, the cells were fixed with 4% paraformaldehyde for 20 min and later stained with crystal violet. Finally, nonmigratory cells were removed from the upper surface of the Transwell membrane.

### RT–PCR

Total RNA was prepared from cultured cells using TRIzol (Invitrogen). cDNA was synthesized from 1 μg of RNA using the SuperScript synthesis system (Invitrogen). The primer sequences used for RT–PCR were as follows: PLD2, 5′-CGAGAAGCTCCTGGTGGTAG-3′ and 5′-CCAGTCCTTGGTGATGAGGT-3′; TRAP, 5′-ACAGCCCCCCACTCCCACCCT-3′ and 5′-TCAGGGTCTGGGTCTCCTTGG-3′; and GAPDH, 5′-ACTTTGTCAAGCTCATTTCC-3′ and 5′-TGCAGCGAACTTTATTGATG-3′.

### Quantitative real-time PCR

Quantitative PCR was performed with an ABI 7500 Real-Time PCR System and SYBR Green dye (Applied Biosystems, Foster City, CA). The following primers were used: PLD2, 5′-CCAGCAAACAGAAATACTTGGAAA-3′ and 5′-GGCGTGGTAATTGCGATAGAA-3′; PLD1, 5′-TTGCTGATTTCATTGACAGGTACTC-3′ and 5′-CATGGACCACAGAGCCAATATC-3′; Atp6v0d2, 5′-GAGCTGTACTTCAATGTGGACCAT-3′ and 5′-CTGGCTTTGCATCCTCGAA-3′; DC-STAMP, 5′-CTTCCGTGGGCCAGAAGTT-3′ and 5′-AGGCCAGTGCTGACTAGGATGA-3′; c-Fos, 5′-AGGCCCAGTGGCTCAGAGA-3′ and 5′-GCTCCCAGTCTGCTGCATAGA-3′; NFATc1, 5′-ACCACCTTTCCGCAACCA-3′ and 5′-TTCCGTTTCCCGTTGCA-3′; TRAP, 5′-TCCCCAATGCCCCATTC-3′ and 5′-CGGTTCTGGCGATCTCTTTG-3′; and MMP-9, 5′-AAAGACCTGAAAACCTCCAACCT-3′ and 5′-GCCCGGGTGTAACCATAGC-3′.

### Western blotting and immunoprecipitation

BMMs or osteoclasts were washed with PBS and lysed with lysis buffer [50 mM Tris-HCl (pH 7.4), 150 mM NaCl, 1% NP-40, 1 mM EDTA] supplemented with Halt protease/phosphatase inhibitor cocktail (Thermo Scientific Inc., Rockford, IL, USA). First, the protein concentration of the cell lysates was determined using a bicinchoninic acid kit (Pierce, Rockford, IL). Next, an aliquot of protein (40 μg) was subjected to 8% or 10% SDS-PAGE and transferred onto a polyvinylidene difluoride membrane. The membrane was blocked in 5% skim milk or 1% BSA and immunoblotted with specific primary antibodies. Finally, immunoreactivity was quantified using an ECL-Plus detection kit (Amersham Pharmacia Biotech, Piscataway, NJ, USA). For immunoprecipitation, cell lysates were incubated with anti-PLD2 antibody or control IgG followed by Sepharose A beads (GE Healthcare). The immunoprecipitated proteins were separated by 8% SDS-PAGE and immunoblotted as described above.

### Immunofluorescence and actin ring staining

BMMs were seeded on glass slides in 24-well plates and cultured with M-CSF and RANKL for 4–5 days. Then, the cells were fixed with 4% paraformaldehyde, permeabilized with 0.1% Triton X-100 and blocked in 0.2% BSA. The cells were then incubated with anti-tubulin or anti-acetylated tubulin primary antibodies. After washing with PBS, the samples were incubated with secondary antibodies and mounted with 80% glycerol in PBS. For actin ring staining, F-actin was labeled with TRITC-conjugated phalloidin (Sigma) or Alexa Fluor 488 phalloidin (Invitrogen), and the nuclei were stained with Hoechst 33258 (Sigma). The samples were visualized using a fluorescence microscope (Leica Microsystems, Germany).

### Resorption pit assay

BMMs were cultured on bone slices in the presence of M-CSF and RANKL. After 5 days, the cultured osteoclasts were removed from bone slices through mechanical agitation. The bone slices were then incubated with peroxidase-conjugated wheat germ agglutinin (Sigma) and stained with 3,3′-diaminobenzidine (Sigma). The resorbed pit area and relative pit size were measured by a Java-based image analysis program (ImageJ).

### Statistics

Statistical analyses of all experiments were performed using Student’s *t* test in Microsoft Excel 2016 (Microsoft, USA). Differences for which *p* < 0.05 were considered statistically significant, and the data are presented as the mean ± standard deviation (SD).

## Results

### *Pld2-*deficient mice exhibit a low bone mass

**Fig. 1:**
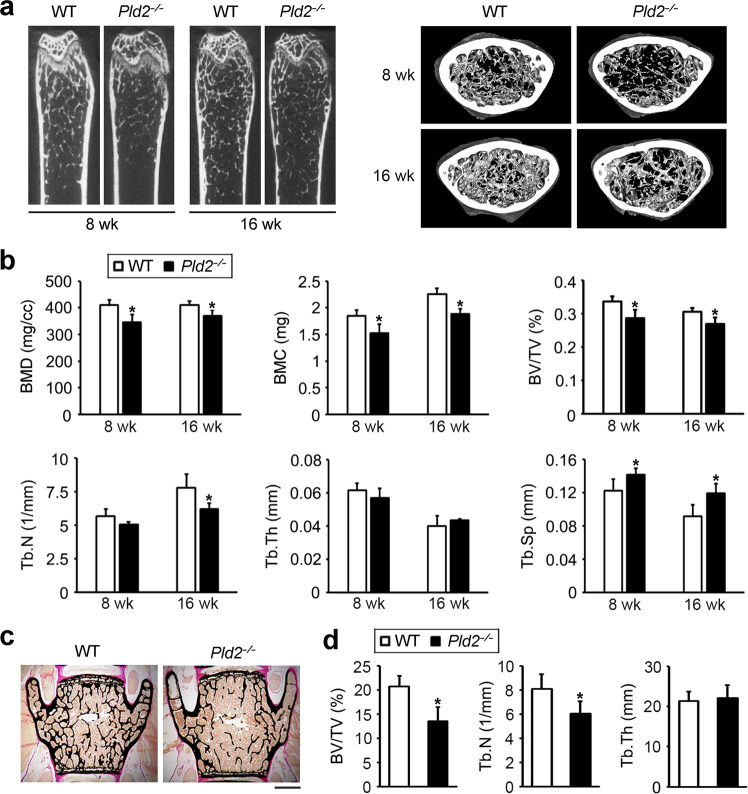
*Pld2*^*−*/*−*^ mice exhibit decreased bone mass in the femur and lumbar vertebrae. **a** Microcomputed tomography (micro-CT) images of distal femurs from 8- or 16-week-old WT and *Pld2*^*−*/*−*^ mice. **b** Quantitative micro-CT analysis of trabecular bone parameters including bone mineral density (BMD), bone mineral content (BMC), bone volume per tissue volume (BV/TV), trabecular number (Tb.N), trabecular thickness (Tb.Th), and trabecular separation (Tb.Sp). Data are expressed as the mean ± SD. *p < 0.05 versus WT; 8-week-old mice, *n* = 5; 16-week-old mice, *n* = 4. **c** Von Kossa staining of vertebral sections from 16-week-old WT and *Pld2*^*−*/*−*^ mice. Scale bar, 50 μm. **d** Histomorphometric quantification of the data from **c**. Data are expressed as the mean ± SD. *p < 0.05 versus WT; *n* = 4.

To evaluate the physiological role of PLD2 in bone homeostasis, the femurs of 8- and 16-week-old *Pld2*^*−/−*^ mice were analyzed with a micro-CT scanner. The *Pld2*^*−/−*^ mice at both ages displayed a significant decrease in bone mineral density, bone mineral content, and bone volume per tissue volume (BV/TV) compared to those of their wild-type (WT) littermates (Fig. 1a, b). Trabecular number (Tb.N) was decreased in the *Pld2*^*−/−*^ mice, with a related increase in trabecular separation (Th.Sp). No difference in trabecular thickness (Tb.Th) was observed between *Pld2*^*−/−*^ mice and their WT counterparts (Fig. 1b). Additionally, the low bone mass phenotype was further confirmed by nondecalcified bone histology. Von Kossa staining of lumbar vertebrae sections showed a significant reduction in BV/TV and Tb.N without a change in Tb.Th in the *Pld2*^*−/−*^ mice compared to WT littermates (Fig. 1c, d), as was the case upon analysis of the femurs.

**Fig. 2:**
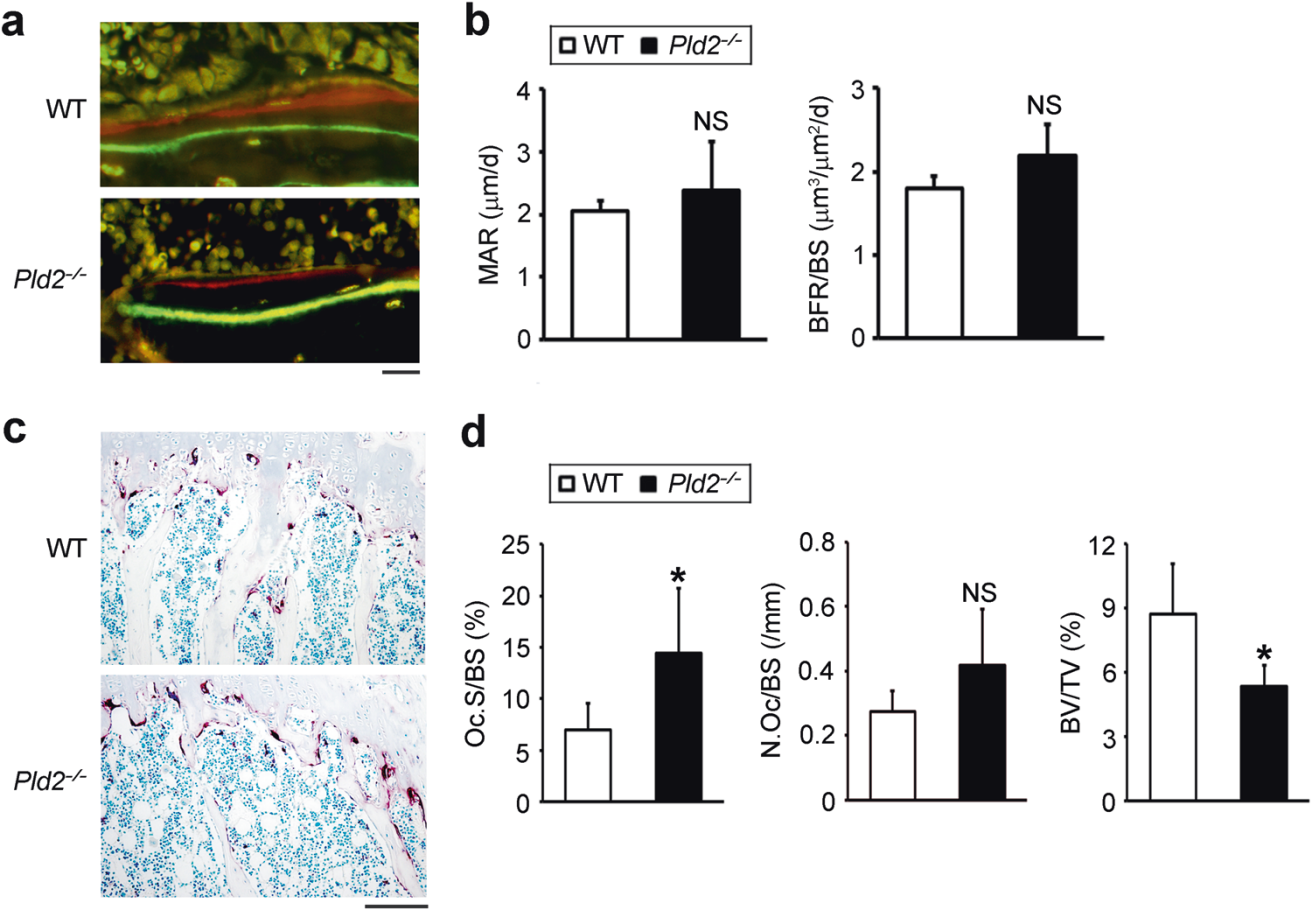
Deletion of *Pld2* increases the osteoclast resorptive surface without altering bone formation. **a** Calcein and alizarin red double-labeling images of trabecular bones in proximal tibia from 8-week-old WT and *Pld2*^*−*/*−*^ mice. Scale bar, 20 μm. **b** Mineral apposition rate (MAR) and bone formation rate (BFR/BS) measured using histomorphometry of the images in **a**. NS, not significant; *n* = 6. **c** TRAP staining images in proximal tibia from 8-week-old WT and *Pld2*^*−*/*−*^ mice. Scale bar, 100 μm. **d** Osteoclast surface per bone surface (Oc.S/BS), number of osteoclasts per bone surface (N.Oc/BS), and BV/TV in **c**. Data are expressed as the mean ± SD. *p < 0.05 versus WT; *n* = 5.

The osteopenic phenotype of the *Pld2*^*−/−*^ mice could be related to decreased bone formation and/or increased bone resorption. To address this issue, we also performed histological analysis of bone tissues. Dynamic histomorphometry of calcein green- and alizarin red-labeled tibia sections showed no differences in the bone formation parameters such as mineral apposition rate (MAR) and bone formation rate (BFR) between *Pld2*^*−/−*^ mice and WT littermates (Fig. 2a, b). In contrast, the resorptive bone surface area was larger in *Pld2*^*−/−*^ mice than in WT mice (Fig. 2c, d). Osteoclast surface per bone surface (Oc.S/B.S) was significantly increased in *Pld2*^*−/−*^ mice compared to WT controls, as indicated by TRAP staining. However, there was no difference in the osteoclast number per bone surface (N.Oc/BS) (Fig. 2c, d).

### Deletion of *Pld2* increases osteoclast spreading and bone resorption

**Fig. 3:**
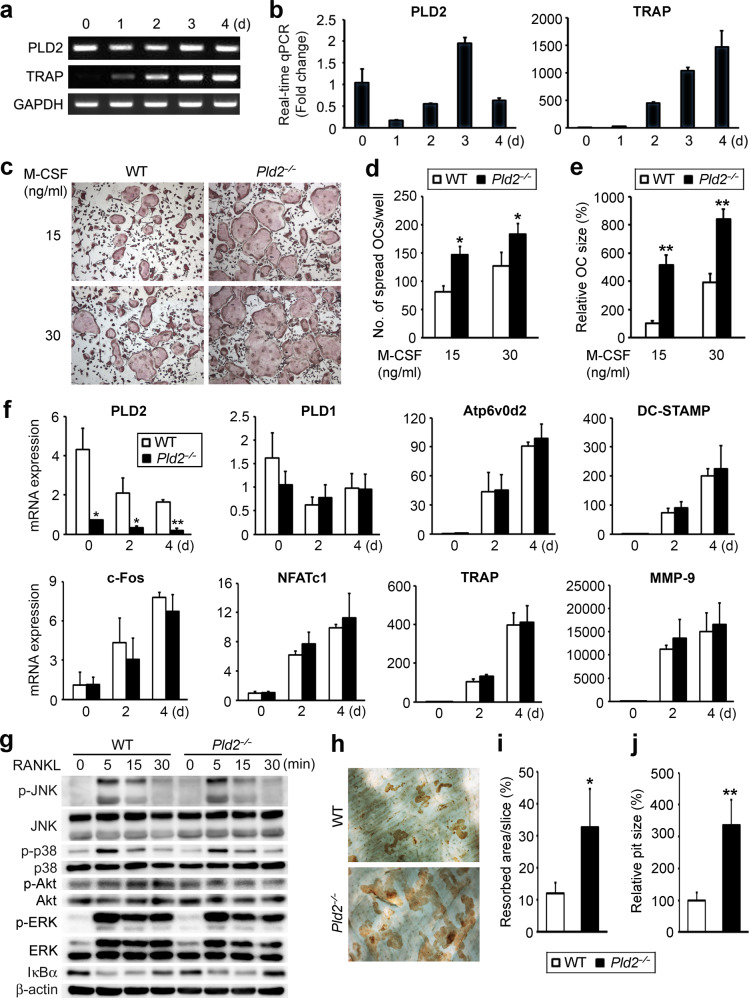
*Pld2* deficiency promotes osteoclast spreading and bone resorption. **a**, **b** BMMs were cultured with RANKL (20 ng/ml) and M-CSF (30 ng/ml) for the indicated number of days. PLD2 expression was assessed using RT–PCR (**a**) and real-time PCR (**b**). TRAP served as a positive control for osteoclastogenesis. **c**–**e** WT and *Pld2*^*−*/*−*^ BMMs were cultured with M-CSF (15 or 30 ng/ml) and RANKL (20 ng/ml) for 4 days. **c** Cultured cells were stained for TRAP. **d** Quantification of spread osteoclast (OC) number. **e** Quantification of relative OC size. **f** WT and *Pld2*^*−*/*−*^ BMMs were cultured in osteoclastogenic medium for the indicated days, and expression of the indicated genes was analyzed by real-time PCR. **g** Serum-starved WT and *Pld2*^*−*/*−*^ BMMs were treated with RANKL (50 ng/ml). Immunoblotting of total protein extracts was performed with the indicated antibodies. **h**–**j** WT and *Pld2*^*−*/*−*^ BMMs were cultured on bone slices with M-CSF and RANKL. **h** After 5 days, the bone slices were stained with peroxidase-conjugated wheat germ agglutinin. **i** Quantification of the resorbed pit area. **j** Quantification of relative pit size. Data are expressed as the mean ± SD. *p < 0.05, **p < 0.001.

Having established that *Pld2* deficiency decreases bone mass due to accelerated osteoclast development, we turned to in vitro osteoclast culture and first examined the expression pattern of PLD2 during osteoclastogenesis. PLD2 expression was abundant in osteoclast precursors (BMMs), rapidly decreased within 1 day after the addition of RANKL and then increased until day 3 of culture (Fig. 3a, b). Next, we examined the impact of *Pld2* deficiency on osteoclast formation. BMMs derived from *Pld2*^*−/−*^ mice and WT littermates were cultured with RANKL and two different concentrations of M-CSF. Interestingly, the number of well-spread osteoclasts was significantly higher in cultures isolated from *Pld2*^*−/−*^ osteoclasts than in WT counterparts at both M-CSF concentrations (Fig. 3c, d). Additionally, the relative osteoclast size was much greater in *Pld2*^*−/−*^ cultures than in WT controls (Fig. 3e).

To determine whether the osteoclast phenotype in the absence of *Pld2* reflects improved osteoclast fusion and/or differentiation, we assessed the expression of relevant osteoclastogenic indicators. *Pld2* deletion had no impact on the mRNA expression of osteoclast fusion markers (Atp6v0d2 and DC-STAMP) or various osteoclast differentiation markers (Fig. 3f). These observations were also confirmed at the protein level by immunoblotting (data not shown). Furthermore, RANKL-mediated activation of signaling molecules required for osteoclastogenesis was unaltered in the absence of *Pld2* (Fig. 3g). These results demonstrate that *Pld2* deficiency does not affect osteoclast fusion or differentiation induced by RANKL.

Given that *Pld2* depletion does not alter osteoclastogenesis, we next investigated the impact of *Pld2* deficiency on osteoclastic bone resorption. Mature osteoclasts from WT and *Pld2*^*−/−*^ mice were generated on bone slices. After 5 days, the resorption pits were visualized with peroxidase-conjugated wheat germ agglutinin staining. Consistent with their super spread morphology, *Pld2*-deficient osteoclasts significantly enhanced resorption lacuna formation (Fig. 3h, i). In addition to the increased total resorption area, the average pit size was approximately three times larger in *Pld2*^*−/−*^ osteoclasts (Fig. 3j).

### PLD2 regulates the proliferation and migration of osteoclast lineage cells

**Fig. 4:**
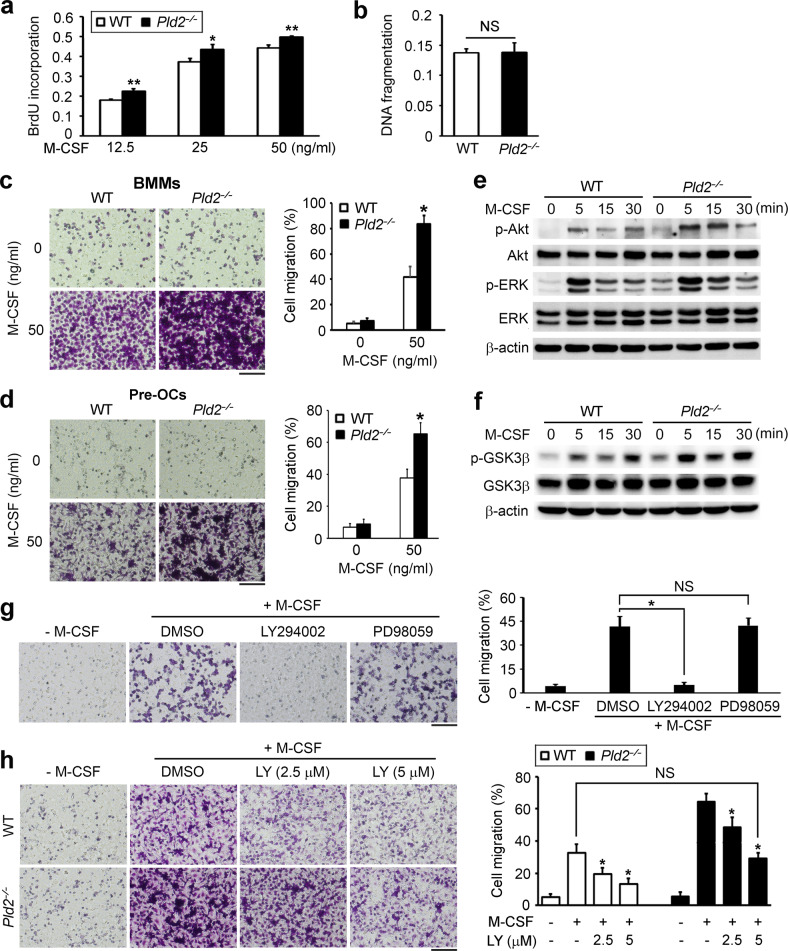
*Pld2* deficiency accelerates the migration of osteoclast lineage cells by activating M-CSF-induced Akt signaling. **a** WT and *Pld2*^*−*/*−*^ BMMs were cultured with M-CSF at the indicated concentrations. After 3 days, BrdU incorporation was measured. **b** WT and *Pld2*^*−*/*−*^ BMMs were cultured with M-CSF (30 ng/ml). The extent of apoptosis was measured using cell death ELISA. **c**, **d** Cytokine-starved BMMs (**c**) or pre-OCs (**d**) from WT and *Pld2*^*−*/*−*^ mice were subjected to migration assays after treatment with M-CSF. Representative images of migrated cells stained with crystal violet (left) and percentage of migrated cells (right). **e**, **f** Cytokine-starved WT and *Pld2*^*−*/*−*^ BMMs were stimulated with M-CSF (50 ng/ml) for the indicated times. Immunoblotting was used to assess the phosphorylation of Akt and ERK (**e**) and GSK3β (**f**); total Akt, ERK, and GSK3β served as loading controls. **g** Cytokine-starved WT BMMs were treated with or without LY294002 (10 μM) or PD98059 (10 μM) for 16 h, and then a migration assay toward M-CSF (50 ng/ml) was performed. Right panel, percentage of migrated cells. **h** Cytokine-starved BMMs from WT and *Pld2*^*−*/*−*^ mice were treated with or without LY294002 (LY, 2.5 or 5 μM) for 16 h, and then a migration assay was performed. Right panel, percentage of migrated cells. Data are expressed as the mean ± SD. *p < 0.05. All scale bars represent 100 μm.

The proliferation, survival, and migration of osteoclast lineage cells are also key to osteoclast formation and function. Therefore, we performed a BrdU incorporation assay and cell death ELISA to examine whether *Pld2* deficiency affects the proliferation and/or apoptosis of osteoclast precursor cells (BMMs). The analysis of BrdU incorporation showed that *Pld2* deficiency slightly increased cell proliferation in response to M-CSF at various concentrations (Fig. 4a). In contrast, PLD2 had no impact on BMM survival, as dictated by cell death detection via ELISA (Fig. 4b).

We next investigated the role of PLD2 in cell migration using the Transwell culture system. The depletion of *Pld2* strongly promoted M-CSF-induced migration of both types of BMMs (Fig. 4c) and preosteoclasts at day 2 (Fig. 4d). M-CSF activates the PI3K/Akt and Grb2/ERK cascades in osteoclastic cells. Therefore, we assessed whether Akt or ERK signaling is altered in the absence of *Pld2*. The phosphorylation levels of Akt and ERK were strongly increased in *Pld2*-deficient cells after M-CSF exposure (Fig. 4e). Generally, PI3K–Akt regulates cell growth, migration, and survival through GSK3β. Activated Akt phosphorylates and inhibits GSK3β, thereby stimulating these cellular functions. Therefore, we evaluated the phosphorylation level of GSK3β and found that GSK3β was hyperphosphorylated in *Pld2*^*−/−*^ BMMs (Fig. 4f). The accelerated migration in *Pld2*^*−/−*^ cells in response to M-CSF reflects activation of the PI3K–Akt and/or Grb2–ERK pathway. To determine which signaling pathway is responsible for the M-CSF-induced increase in osteoclastic cell migration, we used inhibitors of these pathways. Transwell migration assays showed that a PI3K inhibitor (LY294002), but not an ERK inhibitor (PD98059), strongly downregulated the capacity of M-CSF to promote migration in WT precursor cells (Fig. 4g). Based on our observation that PI3K–Akt regulates osteoclastic cell migration, we then treated WT and *Pld2*^*−/−*^ BMMs with a PI3K inhibitor. The inactivation of PI3K in WT BMMs dose-dependently suppressed cell migration (Fig. 4h). Importantly, *Pld2*-deficient BMMs were more resistant to the suppressive effect of the PI3K inhibitor on cell migration (Fig. 4h). Compared to WT BMMs, *Pld2*^*−/−*^ BMMs migrated at normal levels in the presence of 5 μM LY294002. Thus, M-CSF-mediated activation of the PI3K–Akt pathway is most likely responsible for the enhanced migration of *Pld2*^*−/−*^ osteoclast lineage cells.

### *Pld2* deficiency accelerates cytoskeletal organization in osteoclasts

**Fig. 5:**
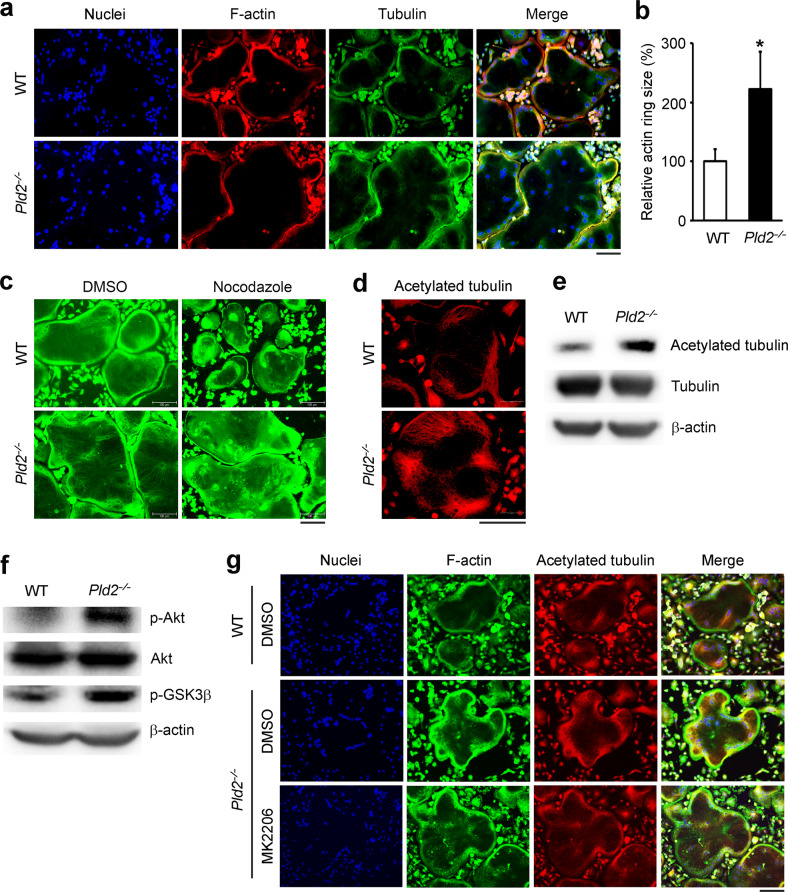
Deletion of *Pld2* promotes actin ring formation and microtubule acetylation through Akt activation. **a**–**d** WT and *Pld2*^*−*/*−*^ BMMs were cultured on coverslips for 5 days with M-CSF and RANKL. **a** Nuclei, F-actin, and tubulin in osteoclasts were stained with Hoechst (blue), TRITC-conjugated phalloidin (red), and anti-tubulin (green) antibodies, respectively, and detected using a fluorescence microscope. **b** Quantification of relative actin ring size. Data are expressed as the mean ± SD. *p < 0.05. **c** Osteoclasts were treated without or with nocodazole (1 μM) for 30 min, fixed, and labeled with anti-tubulin antibody. **d** Osteoclasts were stained with an anti-acetylated tubulin antibody. **e**, **f** Osteoclast lysates were immunoblotted with the indicated antibodies. **g** Osteoclasts were cultured without or with the Akt inhibitor MK2206 (1 μM) for 16 h, fixed, and labeled with FITC-conjugated phalloidin (green) and anti-acetylated tubulin (red) antibody. All scale bars represent 100 μm.

The tendency of *Pld2*-deficient osteoclasts to spread (Fig. 3c–e) suggests the accelerated organization of their actin cytoskeleton. At the beginning of osteoclastogenesis, the actin cytoskeleton is organized into clusters, which are later arranged around the cell periphery to form F-actin rings in mature osteoclasts. The size of actin rings reflects the ability of osteoclasts to resorb bone. To analyze the role of PLD2 in the osteoclast cytoskeleton, we generated WT and *Pld2*^*−/−*^ osteoclasts on glass coverslips and stained the F-actin rings with TRITC-conjugated phalloidin. Immunostaining data revealed that compared to WT cells, *Pld2*-deficient osteoclasts displayed larger actin ring formation (Fig. 5a). The relative size of the actin rings was approximately twofold larger in *Pld2*^*−/−*^ osteoclasts than in WT polykaryons (Fig. 5b).

Actin ring formation in osteoclasts depends on an intact microtubule network. To examine the impact of *Pld2* depletion on microtubule organization, we applied nocodazole, a microtubule-depolymerizing agent. While nocodazole treatment resulted in significant disruption of the microtubule cytoskeleton in WT osteoclasts, *Pld2*-deficient osteoclasts had a more nocodazole-resistant population of stable microtubules (Fig. 5c). The acetylation of microtubules in mature osteoclasts represents microtubule stability, which led to our assessment of microtubule acetylation in *Pld2*^*−/−*^ and WT osteoclasts. Immunofluorescence staining using an anti-acetylated tubulin antibody showed that *Pld2*^*−/−*^ osteoclasts possessed more abundant and highly acetylated tubulin than WT cells (Fig. 5d). These observations were further confirmed by immunoblotting in the same cells (Fig. 5e).

The Akt–GSK3β signaling axis is pivotal for microtubule stability in osteoclasts^19^. Therefore, we examined this key function and found that Akt phosphorylation was strongly enhanced in *Pld2*^*−/−*^ osteoclasts (Fig. 5f). Consistently, GSK3β phosphorylation was increased in *Pld2*^*−/−*^ osteoclasts. Next, we applied the Akt-specific inhibitor MK2206 to attenuate Akt activity in *Pld2*^*−/−*^ osteoclasts. As shown in Fig. 5g, the increased microtubule acetylation in the absence of *Pld2* was restored by the Akt inhibitor, indicating that *Pld2* deficiency promotes microtubule stabilization by increasing Akt activity.

### PLD2 forms a complex with c-Fms and PI3K

**Fig. 6:**
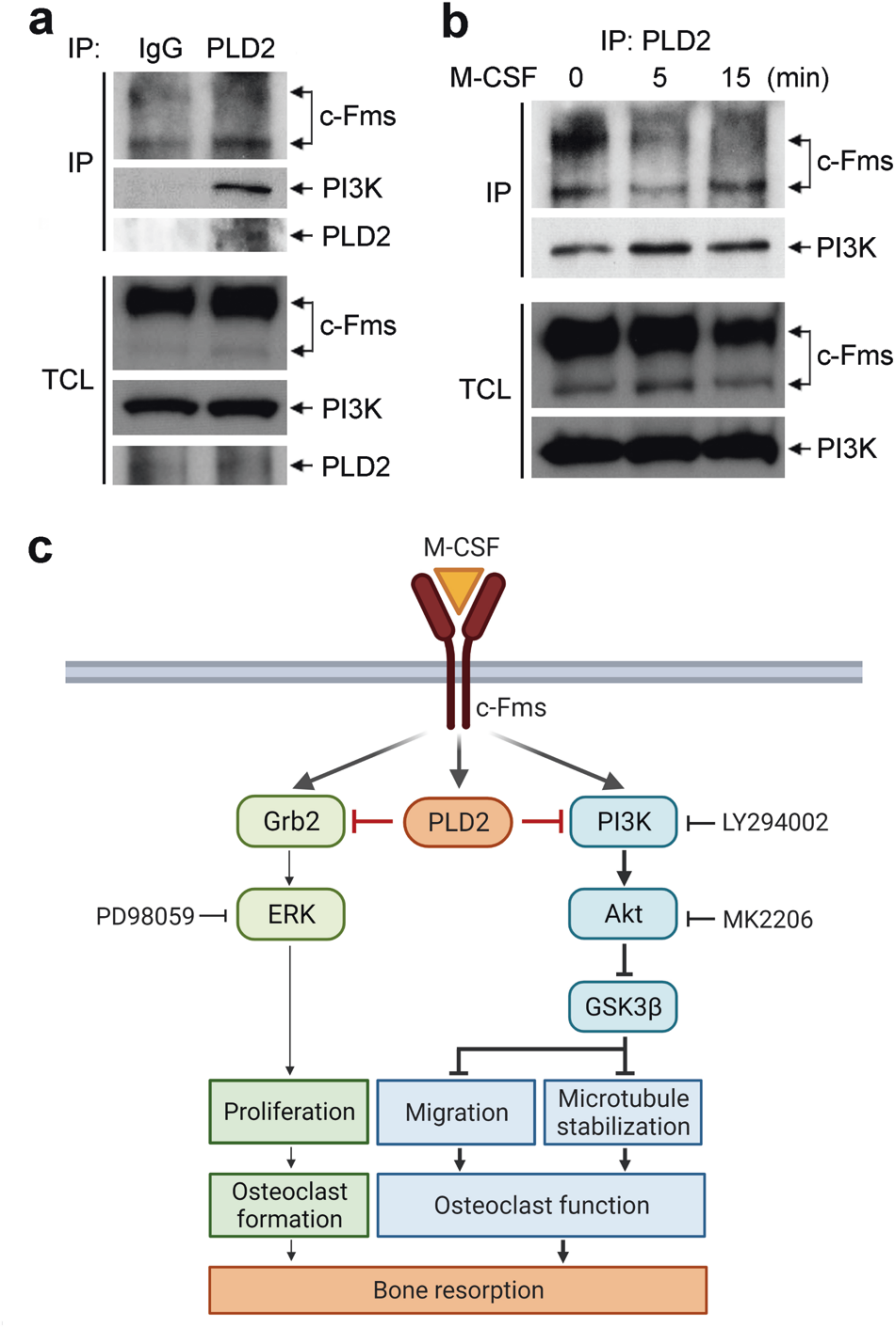
PLD2 interacts with c-Fms and PI3K. **a** Endogenous PLD2 in BMMs was immunoprecipitated and subjected to immunoblotting with the indicated antibodies. Nonspecific proteins were immunoprecipitated using normal mouse IgG. **b** Cytokine-starved BMMs were stimulated with M-CSF (50 ng/ml) for 5 and 15 min and subjected to immunoprecipitation using anti-PLD2 antibody, followed by immunoblotting for the indicated antibodies. TCL, total-cell lysate. **c** Working model of PLD2 as a negative regulator of osteoclastic bone resorption.

Given our data showing that PLD2 specifically regulates the M-CSF-induced PI3K–Akt signaling pathway, we hypothesized that PLD2 can bind the M-CSF receptor, c-Fms, and/or PI3K. Therefore, endogenous PLD2 was immunoprecipitated using an anti-PLD2 or mouse IgG control antibody to address this hypothesis. As shown in Fig. 6a, PLD2 interacted with both c-Fms and PI3K in BMMs. PI3K directly binds c-Fms after M-CSF stimulation^32,33^, resulting in Akt activation. To further investigate the effect of M-CSF on PLD2-c-Fms-PI3K complex formation, cytokine-starved BMMs were treated with M-CSF and immunoprecipitated with an anti-PLD2 antibody. Under basal conditions, PLD2 was associated with both c-Fms and PI3K (Fig. 6b). Interestingly, the binding of PLD2 to c-Fms was reduced by M-CSF stimulation, whereas the interaction between PLD2 and PI3K increased following M-CSF exposure (Fig. 6b). Collectively, our data suggest that the enhanced interaction between PLD2 and PI3K in response to M-CSF may be a key mechanism by which PLD2 controls the PI3K–Akt pathway.

## Discussion

PLD family proteins function as important signaling molecules in various tissues and cellular processes. Numerous animal model studies involving targeted gene disruption and/or pharmacological approaches have suggested that PLD proteins could provide a therapeutic basis for immunological, vascular, and neurological disorders^21,22^. In this study, we investigated the role of PLD2 in the skeleton using *Pld2*^*−/−*^ mice and found that PLD2 plays a critical role in bone metabolism and homeostasis. We also demonstrated that PLD2 negatively controls osteoclast bone resorption by regulating osteoclastic cell migration and microtubule stabilization via the M-CSF-mediated PI3K–Akt–GSK3β axis and partly by modulating precursor cell proliferation via the Grb2–ERK pathway (Fig. 6c).

Our in vivo data reveal that *Pld2*^*−/−*^ mice display a decrease in both trabecular and lumbar vertebral bone mass. The decrease in bone mass in the absence of *Pld2* was associated with increased osteoclast function in vivo. Consistently, in vitro data further confirmed the suppressive role of PLD2 in bone resorptive activity, as indicated by the increased resorption pit formation in *Pld2*-deficient osteoclasts. The accelerated bone resorption was due to increased migration and cytoskeletal organization in *Pld2*^*−/−*^ osteoclasts but not the differentiation and/or fusion of their precursors. Meanwhile, the osteoblast parameters (MAR and BFR) were not significantly changed in the *Pld2*^*−/−*^ mice, indicating that *Pld2* deficiency does not affect bone formation in vivo. Consistent with our findings, a recent study reported that in vitro pharmacological suppression of PLD2 with a chemical inhibitor had no profound effect on the osteoblast mineralization process^25^. Additionally, more recently, our study showed that PLD2 was barely detected during osteoblast differentiation^26^. Thus, in addition to previous studies, our findings reveal that PLD2 does not significantly affect osteoblast bone-forming activity.

Cell migration is a highly active process that involves actin cytoskeleton remodeling^34^. In particular, the migration of osteoclastic cells is crucial for their bone resorption function. Furthermore, PLD2 regulates the migration of diverse cell types, such as macrophages, leukocytes, and vascular smooth muscle cells^35–37^. Based on these studies, we hypothesized that PLD2 can regulate the migration of osteoclast lineage cells. Supporting this hypothesis, we discovered that *Pld2* deficiency increases osteoclastic cell migration in response to M-CSF. The relevance of PLD2 to macrophage migration has also been reported in a previous study^35^. However, unlike our observations, a previous study showed that silencing PLD2 using siRNA reduces M-CSF-mediated cell migration, whereas overexpression of PLD2 has the opposite effect. Furthermore, although the study suggested that PLD2 plays a positive role in the migration of macrophage cells, the experiments were performed using the RAW/LR5 macrophage cell line, which often does not function identically to primary cells. However, we used primary BMMs and preosteoclasts isolated from *Pld2*^*−/−*^ mice in this study. This discrepancy may be due to the differences in cell type and off-target effects of knockdown or the ectopic expression of PLD2.

The activated PI3K–Akt pathway is a key signaling pathway for cell migration, proliferation, survival, and actin remodeling in various cell types. The critical role of PI3K in osteoclast migration and function was well demonstrated by a genetic study^38^. Mice lacking the p85α subunit of PI3K exhibited increased bone mass with defective cell migration and bone resorption, which were attributed to the reduced activation of Akt. Our results demonstrate that *Pld2* deficiency increases Akt activation induced by M-CSF but not RANKL. Thus, enhanced Akt activity by M-CSF contributes to the accelerated migration of osteoclast lineage cells in the absence of *Pld2*.

In addition to cell migration, PLD2 regulates osteoclast cytoskeletal organization. Its deficiency enhances osteoclast spreading, which generally reflects the ability of osteoclasts to form actin rings^18,39–42^. In fact, *Pld2*-deficient osteoclasts possess enlarged actin rings and hyper-resorption. Actin ring organization in osteoclasts is governed by a microtubule network, specifically acetylated tubulin. Consequently, failure to acetylate microtubules increases bone mass due to defective bone resorption^16,19,20^. The acetylation of microtubules, which stabilizes them, is regulated by several factors, including PI3K–Akt. Thus, the PI3K–Akt cascade is critical for cell migration and a key factor for microtubule stabilization. Matsumoto et al. reported the importance of the Akt signaling molecule in actin ring formation and microtubule stability^19^. Treatment with an Akt inhibitor in osteoclasts disrupted actin ring formation with abnormal regulation of acetylated tubulin; however, the expression level of acetylated tubulin was enhanced when catalytically active Akt was overexpressed^19^. The effect of Akt on microtubule stabilization is regulated by GSK3β, an effector signaling molecule downstream of Akt. The inhibition of GSK3β activity by Akt permits the activation of microtubule-associated proteins responsible for microtubule stabilization^19,43^. These findings reveal that Akt promotes osteoclast bone-resorbing activity by regulating actin ring organization and microtubule stability. We demonstrated by immunostaining and immunoblotting that *Pld2* deficiency increases the expression of the acetylated form of tubulin. Moreover, the activation of Akt and consequent inactivation of GSK3β were observed in *Pld2*-deficient osteoclasts. Furthermore, an Akt inhibitor restored tubulin acetylation and actin ring formation in the absence of *Pld2*. However, there was no significant change in actin ring size. This may be due to the short treatment time and low concentration of the Akt inhibitor used in this study. Together, our results indicate that PLD2 controls microtubule stability by regulating the Akt–GSK3β axis. Similar to our findings, a group working on mast cell function showed that PLD2 regulates dynamic microtubule rearrangement during mast cell activation^24^. Importantly, microtubule formation and the FcεRI-mediated activation of Akt were increased in mast cells isolated from *Pld2*^*−/−*^ mice, showing that PLD2 contributes to the negative regulation of cytoskeletal organization in mast cells.

In summary, we have identified PLD2 as a negative regulator of osteoclast function, specifically M-CSF signaling, and is essential for osteoclastic cell migration and cytoskeletal organization. PLD2 binds the M-CSF receptor (c-Fms) and PI3K and antagonizes osteoclast migration and microtubule stabilization by attenuating the Akt–GSK3β signaling axis, leading to impaired bone resorption. Therefore, the modulation of PLD2 may provide a novel strategy for treating skeletal diseases, including osteoporosis and rheumatoid arthritis.

## References

[1] Boyle WJ, Simonet WS, Lacey DL (2003). Osteoclast differentiation and activation. Nature.

[2] Choi JY (2020). Healthy bone tissue homeostasis. Exp. Mol. Med..

[3] Takayanagi H (2007). Osteoimmunology: shared mechanisms and crosstalk between the immune and bone systems. Nat. Rev. Immunol..

[4] Teitelbaum SL (2000). Bone resorption by osteoclasts. Science.

[5] Kong YY (1999). OPGL is a key regulator of osteoclastogenesis, lymphocyte development and lymph-node organogenesis. Nature.

[6] Lacey DL (1998). Osteoprotegerin ligand is a cytokine that regulates osteoclast differentiation and activation. Cell.

[7] Wong BR (1997). TRANCE (tumor necrosis factor [TNF]-related activation-induced cytokine), a new TNF family member predominantly expressed in T cells, is a dendritic cell-specific survival factor. J. Exp. Med..

[8] Yasuda H (1998). Osteoclast differentiation factor is a ligand for osteoprotegerin/osteoclastogenesis-inhibitory factor and is identical to TRANCE/RANKL. Proc. Natl Acad. Sci. USA.

[9] Feng X, Teitelbaum SL (2013). Osteoclasts: new Insights. Bone Res..

[10] Mun SH, Park PSU, Park-Min KH (2020). The M-CSF receptor in osteoclasts and beyond. Exp. Mol. Med..

[11] Teitelbaum SL (2011). The osteoclast and its unique cytoskeleton. Ann. N. Y. Acad. Sci..

[12] Yoshida H (1990). The murine mutation osteopetrosis is in the coding region of the macrophage colony stimulating factor gene. Nature.

[13] Destaing O, Saltel F, Geminard JC, Jurdic P, Bard F (2003). Podosomes display actin turnover and dynamic self-organization in osteoclasts expressing actin-green fluorescent protein. Mol. Biol. Cell.

[14] Jurdic P, Saltel F, Chabadel A, Destaing O (2006). Podosome and sealing zone: specificity of the osteoclast model. Eur. J. Cell Biol..

[15] Destaing O (2005). A novel Rho-mDia2-HDAC6 pathway controls podosome patterning through microtubule acetylation in osteoclasts. J. Cell Sci..

[16] Gil-Henn H (2007). Defective microtubule-dependent podosome organization in osteoclasts leads to increased bone density in Pyk2(-/-) mice. J. Cell Biol..

[17] Guimbal S (2019). Dock5 is a new regulator of microtubule dynamic instability in osteoclasts. Biol. Cell.

[18] Hong JM (2011). Calpain-6, a target molecule of glucocorticoids, regulates osteoclastic bone resorption via cytoskeletal organization and microtubule acetylation. J. Bone Miner. Res..

[19] Matsumoto T (2013). Regulation of bone resorption and sealing zone formation in osteoclasts occurs through protein kinase B-mediated microtubule stabilization. J. Bone Miner. Res..

[20] Zalli D (2016). The actin-binding protein cofilin and its interaction with cortactin are required for podosome patterning in osteoclasts and bone resorption in vivo and in vitro. J. Bone Miner. Res..

[21] Brown HA, Thomas PG, Lindsley CW (2017). Targeting phospholipase D in cancer, infection and neurodegenerative disorders. Nat. Rev. Drug Discov..

[22] Ghim J, Chelakkot C, Bae YS, Suh PG, Ryu SH (2016). Accumulating insights into the role of phospholipase D2 in human diseases. Adv. Biol. Regul..

[23] Frohman MA (2015). The phospholipase D superfamily as therapeutic targets. Trends Pharmacol. Sci..

[24] Zhu M (2015). Differential roles of phospholipase D proteins in FcepsilonRI-mediated signaling and mast cell function. J. Immunol..

[25] Abdallah D (2019). Effects of phospholipase D during cultured osteoblast mineralization and bone formation. J. Cell. Biochem..

[26] Kang DW (2021). Deletion of phospholipase D1 decreases bone mass and increases fat mass via modulation of Runx2, beta-catenin-osteoprotegerin, PPAR-gamma and C/EBPalpha signaling axis. Biochim. Biophys. Acta Mol. Basis Dis..

[27] Sylvia VL (2001). Regulation of phospholipase D (PLD) in growth plate chondrocytes by 24R,25-(OH)2D3 is dependent on cell maturation state (resting zone cells) and is specific to the PLD2 isoform. Biochim. Biophys. Acta.

[28] Tokuda H (1996). Function of Ca2+ in phosphatidylcholine-hydrolyzing phospholipase D activation in osteoblast-like cells. Bone.

[29] Ghim J (2014). Endothelial deletion of phospholipase D2 reduces hypoxic response and pathological angiogenesis. Arterioscler. Thromb. Vasc. Biol..

[30] Lim KE (2015). Core binding factor beta of osteoblasts maintains cortical bone mass via stabilization of Runx2 in mice. J. Bone Miner. Res..

[31] Takeshita S, Kaji K, Kudo A (2000). Identification and characterization of the new osteoclast progenitor with macrophage phenotypes being able to differentiate into mature osteoclasts. J. Bone Miner. Res..

[32] Lee AW, States DJ (2000). Both src-dependent and -independent mechanisms mediate phosphatidylinositol 3-kinase regulation of colony-stimulating factor 1-activated mitogen-activated protein kinases in myeloid progenitors. Mol. Cell. Biol..

[33] Xiong Y (2011). A CSF-1 receptor phosphotyrosine 559 signaling pathway regulates receptor ubiquitination and tyrosine phosphorylation. J. Biol. Chem..

[34] Ridley AJ (2003). Cell migration: integrating signals from front to back. Science.

[35] Knapek K (2010). The molecular basis of phospholipase D2-induced chemotaxis: elucidation of differential pathways in macrophages and fibroblasts. Mol. Cell. Biol..

[36] Lehman N (2006). Phagocyte cell migration is mediated by phospholipases PLD1 and PLD2. Blood.

[37] Wang Z (2019). Phosphatidic acid generated by PLD2 promotes the plasma membrane recruitment of IQGAP1 and neointima formation. FASEB J..

[38] Munugalavadla V (2008). The p85alpha subunit of class IA phosphatidylinositol 3-kinase regulates the expression of multiple genes involved in osteoclast maturation and migration. Mol. Cell. Biol..

[39] Kim HJ, Lee DK, Jin X, Che X, Choi JY (2020). Oleoylethanolamide exhibits GPR119-dependent inhibition of osteoclast function and GPR119-independent promotion of osteoclast apoptosis. Mol. Cells.

[40] Kim HJ (2016). G protein-coupled receptor 120 signaling negatively regulates osteoclast differentiation, survival, and function. J. Cell. Physiol..

[41] Kim HJ (2006). Glucocorticoids suppress bone formation via the osteoclast. J. Clin. Invest..

[42] McHugh KP (2000). Mice lacking beta3 integrins are osteosclerotic because of dysfunctional osteoclasts. J. Clin. Invest..

[43] Hur EM (2011). GSK3 controls axon growth via CLASP-mediated regulation of growth cone microtubules. Genes Dev..

